# Supplementary material for: Chromothripsis during telomere crisis is independent of NHEJ, and consistent with a replicative origin
Source: Genome Res. 2019 May;29(5):737–49. doi: 10.1101/gr.240705.118 (PMC6499312; doi:10.1101/gr.240705.118)
Supplement: Supplemental Material [file supp_gr.240705.118_Supplemental_file_1.zip › contigs/annotated_contigs/DB57/contig.3.DB57_length_904_mean_cov_7.97123893805.docx]

**DB57_length_904_mean_cov_7.97123893805**

```
ACAAGAAAATATATGCAATCTAAACACCTAACAAAAGAA|TAATAACCAGAACCTAAAACGAACACAAATAACTTTACCAGAAAAAAAC
                                        >chr6:99657418-99657790 + E=4e-80
AAATAACCAAAAAAAAAAGTAGGCAAAGGATATGAACAGACACTTCTCAAAAGAAGACATTTATGGGGCCAACAAACATGAAAAAAAGC

TCATTATTACTGGTCATTAGAGAAATGCAAATCAAAACCACAATGAGATACCATCTCATGACAGTTAGAATGGCAATCATTAAAAAGTC

AGGAAACAACAGATGCTGGAGAGGATGTGGAGAATTAGGAACACTTTTCACTGTTGGTAGGAGTGTAAATTAGTTCAACCATTGTGGAA

GACAGTGTGACAATTCCTCAAGGATCTAGAACCAGAAATACCATTTGGCCCAGC|AAATGGTATTTTGCTGGAATATTT|GCATATACT
                                                                               >chr4:14
GGGTATATATCCAAA|GGA|GAGTTCTACTGCAGAGTCCTGATGATTTAGATGTACCAATGTTTGAATCCTGTCTCAAAATAGAAAGAG
5166505-145166532 - E=2e-01 >chr8:24462583-24463030 + E=6e-254
AAAACCAGGCATGGTACAATTTATGTGTCAATGGTAGTGATGCTATTCCTACATCTAACACCAACTGTGTAAAATATTGACGTCGCATA

CAGATGTTTCACCTTGTGGTCTCAAAACTCTTTCTAGCTGATAAGTTTAGCAAAATAAATATGTTATCTTCCCTAGATTATGACAGGGC

ACCCAAAACTAAACTACCCTCTCTGGTGATTAAAGGGTTTGAGAAAATAGCATGCTGGTCTTTCTTTTTCAGCAGACAAGGGCAATCAT

ATTAAAAAAATTAAAAATTCCACAAAATTTATTTAGTTTGTATATTTTTTACTTTCAGAAAACAAATGGCAGAAAGGAGGTGCCCCACC

TGACATTACTGAGGTGTTA
```
